# An International Comparison of the Involvement of Epidemiology in the Most Frequently Cited Publications in the Field of Clinical Medicine

**DOI:** 10.2188/jea.11.41

**Published:** 2007-11-30

**Authors:** Ken Takahashi, Masakazu Washio, Aiguo Ren, Noritaka Tokui, Tar Ching Aw, Otto Wong

**Affiliations:** 1 Department of Environmental Epidemiology, University of Occupational and Environmental Health, Kitakyushu, Japan.; 2 Department of Public Health, Faculty of Medicine, Kyushu University, Fukuoka, Japan.; 3 Department of Clinical Epidemiology, University of Occupational and Environmental Health, Kitakyushu, Japan.; 4 Institute of Occupational Health, University of Birmingham, Birmingham, UK.; 5 Applied Health Sciences, Inc., San Mateo, CA, USA.; 6 Department of Epidemiology, Tulane University Medical Center, New Orleans, USA.

**Keywords:** epidemiology, international comparison, evidence-based, bibliometrics

## Abstract

The objectivity, validity and credibility of research in clinical medicine can be enhanced by the appropriate involvement of epidemiology. However, the overall contribution of epidemiology to clinical research, either as a methodology or as a resource for research, has been poorly quantified. We therefore assessed the involvement of epidemiology in influential publications in the field of clinical medicine, and made an international comparison on a quantitative basis. The 500 most frequently cited papers published during 1981-96 in the field of clinical medicine in the US, the UK, and Japan were compared in terms of epidemiological involvement using predetermined criteria. The three criteria were based on the indexing of relevant MeSH keywords, publication types, or the departmental affiliations of the authors. For all three criteria, the proportion of clinical papers with epidemiological involvement was the highest in the US, followed by the UK, whereas it was the lowest in Japan. The difference was almost four-fold between the US and Japan. There was also an increasing trend of epidemiological involvement in publications of clinical medicine over the years, which was more apparent in the US than in either the UK or Japan. These findings may reflect inter-country differences in resources as well as in the stance towards evidence-based health sciences.

## INTRODUCTION

Epidemiology has its roots in the investigation of epidemics of infectious diseases in communities. Subsequently, epidemiology has been applied to the study of non-infectious chronic diseases. An example of a simple definition of epidemiology reads as follows: Epidemiology is the branch of medicine concerned with the determination of cause and distribution of diseases in specific populations and its application in the prevention of diseases. Over the years, the definition of epidemiology has been broadened to encompass all phenomena related to health, including, for example, clinical trials.

The objectivity, validity and credibility of research in clinical medicine can be enhanced by consideration and inclusion of epidemiologic principles and procedures. Different “types” of epidemiological study designs have been used for medical research in many subspecialties of medicine. For example, controlled clinical trials are used in the evaluation of drug efficacy, whereas longitudinal cohort studies are used to assess the carcinogenicity of industrial chemicals. However, the overall contribution of epidemiology to clinical research, either as a methodology or as a resource for scientific research has been poorly quantified. The objective of the present investigation is therefore to assess the role of epidemiology in influential papers (judged by the frequency of citation) in the field of clinical medicine, and to make an international comparison (between the US, UK and Japan) on a quantitative basis.

## MATERIALS AND METHODS

A database of the most frequently cited publications in the field of clinical medicine in three countries (US, UK and Japan) over a defined time period was constructed using data compiled by the Institute of Scientific Information, Philadelphia (ISI). We have arbitrarily chosen to include the 500 most frequently cited publications during 1981-96 in each of the three countries.

The criteria and procedure for selecting the most frequently cited publications were as follows. To be included in the selection, a paper must have appeared in one of the journals listed in the Current Contents/Clinical Medicine database, which covers 1,121 of the world’s leading clinical medicine journals^1^^)^ and is generally accept as such. The paper must have been published during 1981-96 and with citations recorded to it during 1981-97. Initially, for each of the 16 years (1981-1996), the 300 most frequently cited papers that have an address in either the US, the UK, or Japan were selected. This procedure generated a total of 4,800 (16×300) entries. As many publications appeared repeatedly in the list of the 300 most frequently cited papers in more than one year, an overall list for the entire 16 years was consolidated by eliminating duplicates. For each paper, a hard copy of the paper in its printed form was retrieved, and the bibliographic information compiled in the Medline database was obtained. Finally papers were grouped by country based on the information on the location and affiliation of the lead (first) author indicated on the hard copies.

The bibliographic information supplied by Medline, specifically Medical Subject Heading (MeSH) keywords (“descriptors” of content) and publication type (“PT”), was used to assess whether epidemiology was involved in the pertinent paper. We also reviewed the departmental affiliations of all authors (as surrogates for their disciplines) with regard to epidemiology. More specifically, the following criteria were applied to assess the involvement of epidemiology in the most frequently cited papers.

1. Content: involved if any of the following MeSH keywords was indexed to the paper: “epidemiology,” “statistics,” “cohort,” “case-control,” “clinical trials,” or “randomized control (or controlled) trials,” supplemented by informed judgment.
2. Publication type: involved if any of the following terms was indexed to the paper: “clinical trial,” “randomized controlled trial,” or “controlled clinical trial.”
3. Affiliation: involved if any of the following terms was included in the affiliation of any author: “epidemiology,” “statistics,” “biostatistics (e.g., biostatistical center),” “biometrics,” “biometry,” “public health (e.g., department of public health or school of public health),” “prevention (e.g., bureau of prevention or cancer prevention division),” “biomathematics,” or “social medicine.”

Only the 500 most frequently cited papers for each country were analyzed for comparison. In ISI’s original database, the total number of citations received from 1981 through 1997 was counted along with the number of expected citations. The expected citation is a baseline number that is unique to the type of publication (original article, review, editorial, etc.) and also unique to the year in which the publication appeared in a particular journal^2^^)^. For example, two original articles published in the same journal published in the same year would have identical expected citation. However, an editorial published in the same journal and in the same year would have a different expected citation figure.

For the purpose of a time-trend analysis, the entire publication period (1981-96) was divided into five consecutive intervals (1981-83, 1984-86, 1987-89, 1990-92, and 1993-96). It should be noted that the number of publications in earlier intervals tended to be higher than that in recent intervals because of the longer period available to receive citations.

The proportion of the most frequently cited papers with epidemiological involvement according to each of the three criteria was compared in pairs among the three countries using the chi-square test (US vs. UK, US vs. Japan, UK vs. Japan). The time-trend analysis of proportion of publications with epidemiological involvement by the content criterion for each country was based on the Cochran-Armitage test. A p-value less than 0.05 was considered statistically significant, 0.05<p<0.1 marginally significant, and p>0.1 non-significant. The original database was converted into an Excel file and all analyses were performed using SAS (version 6.12) on a personal computer.

## RESULTS

The average number of citations received per paper by country is presented in the Table. The number of actual citations per paper was the highest for the US (672.7), the lowest for Japan (156.3), and in-between for the UK (304.1). Some of the most frequently cited US papers received more than 4,000 citations during 1981-97. A similar international pattern was also observed in terms of the number of expected citations per paper.

**Table.** Profile of the most frequently cited publications in the field of clinical medicine in the three countries.

	U.S.A.	U.K.	Japan
Number of citations per article			
Mean (range)	672.7 (415-4309)	304.1 (191-2916)	156.3 (95-1043)
Number of expected citations* per article			
Mean (range)	108.5 (11.0-865.6)	59.7 (5.3-1143.0)	39.8 (2.6-159.0)
Number of publications by publication period			
‘81 thru ‘83	145	150	119
‘84 thru ‘86	139	134	113
‘87 thru ‘89	118	117	113
‘90 thru ‘92	73	79	122
‘93 thru ‘96	25	20	33
Total	500	500	500

* See Methods for definition.

Informed judgment was used to assess the sensitivity and specificity of screening papers for the involvement of epidemiology using Medline information. The criterion based on “publication type” was judged to be 100% sensitive and specific. For the criterion based on “content,” selection by the aforementioned MeSH keywords produced no false-positives (i.e., specificity =100%), but missed several papers with epidemiological involvement (i.e., sensitivity < 100%) based on our judgment of their content by reviewing the papers themselves. In addition to the 198 papers (96 US, 76 UK, 26 Japan) originally identified as having epidemiological involvement using MeSH keywords, a further 19 papers (10 US, 6 UK, 3 Japan) were included based on our review of the contents of these papers. Thus, the sensitivity of the criterion based on MeSH keywords was 198/(198+19) or 91%.

**Figure 1.**
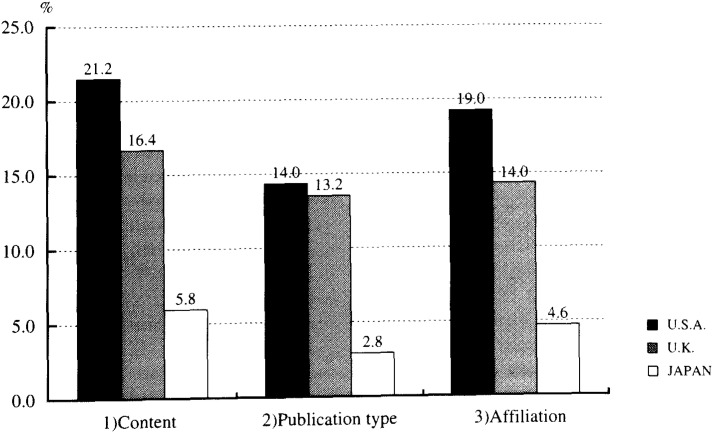
Proportion of publications with epidemiological involvement in the most frequently cited clinical papers in the three countries based on three criteria* (N=500 for each country) P-values for difference in proportion: 1) p=0.052 (U.S.A. vs U.K.), p<0.001 (U.S.A vs Japan), p<0.001 (U.K.vs Japan); 2) n.s. (U.S.A. vs U.K.), p<0.001 (U.S.A. vs Japan), p<0.001 (U.K. vs Japan); 3) p=0.033 (U.S.A. vs U.K.), p<0.001 (U.S.A. vs U.K.), p<0.001 (U.K. vs Japan); n.s.=not significant (p>0.1); *See Methods for criteria.

In Figure 1, the proportion of publications with epidemiological involvement in the most frequently cited clinical papers was compared among the three countries according to each of the three criteria. When the criterion based on content was applied, the proportion of papers with epidemiological involvement was 21.2% (n =106) for the US, 16.4% (n =82) for the UK, and 5.8% (n =29) for Japan. The differences between Japan and the other two countries were statistically significant, while that between the US and the UK was only marginally significant. Based on the criterion of publication type, the respective proportions were 14.0% (n =70) for the US, 13.2% (n =66) for the UK, and 2.8% (n =14) for Japan. Only the differences between Japan and the other two countries reached statistical significance. For the criterion based on affiliation, the respective proportions were 19.0% (n =95) for the US, 14.0% (n =70) for the UK, and 4.6% (n =23) for Japan: all the differences being statistically significant.

Hence, for all three criteria, the proportion of clinical papers with epidemiological involvement was the highest in the US, followed by the UK, and the lowest in Japan. The difference in proportion between the US and the UK was relatively small, whereas those between Japan and the other two countries were much larger in magnitude and consistently significant (p<0.001).

**Figure 2.**
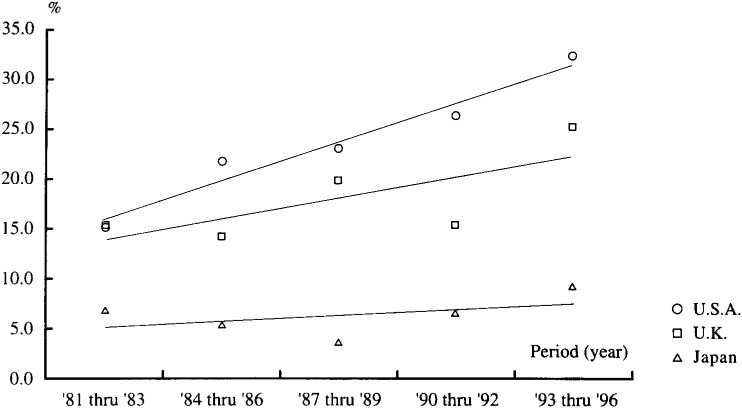
Time trend in the proportion of publications with epidemiological involvement in the most frequently cited clinical papers in the three countries based on the criterion for content* P-values for trend test: p=0.017 (U.S.A), n.s. (U.K.), n.s. (Japan); n.s.=not significant (p>0.1); *See Methods for criterion.

Figure 2 compares the time-trend in proportion of publications with epidemiological involvement in the most frequently cited clinical papers by country according to the criterion based on content. The slope of the regression line was positive for each country, suggesting an increasing trend in the proportion of clinical papers with epidemiological involvement over time. However, the trend was more consistent and statistically significant for US papers only, but fluctuated and not significant for UK or Japanese papers. The slope of the regression line was the largest for the US, smallest for Japan, and in-between for the UK. The regression lines in Figure 2 also demonstrated increasingly wider gaps over time among the three countries in the proportion of most frequently cited clinical papers with epidemiological involvement.

## DISCUSSION

Whether or how the content of a paper is related to a specific discipline such as epidemiology, depends on the definition of the discipline, and in practice, the criteria applied. As the present analyses depended on the use of predefined indices available in the Medline database, the validity and reliability of the indexing procedure need to be discussed. The fact that the indexed information has been designed for the purpose of retrieving papers from the database justified, in part, the use of this method^3^^)^. Furthermore, different criteria for content were applied objectively to further explore this issue.

The narrower criterion for content, which was equivalent to the criterion applied in the current analysis except for exercising human judgment, produced 19.2% (n =96) of US papers, 15.2% (n =76) of UK papers, and 5.2% (n =26) of Japanese papers categorized as having epidemiological involvement, with marginal statistical significant difference between the US and the UK (p =0.094) and statistically significant differences between Japan and the other two countries (p<0.001, respectively). Subsequently, the following descriptors were added to the narrower criterion: “mortality,” “follow-up,” “prevention and control,” “prospective,” “longitudinal,” “retrospective,” “risk factor,” and “statistical.” The application of this broader criterion produced 34.4% (n =172) of US papers, 32.2% (n =161) of UK papers, and 15.0% (n =75) of Japanese papers to be categorized as having epidemiological involvement: with no statistically significant difference between the US and the UK, but with statistically significant differences between Japan and the other two countries. Although these new terms are perhaps more general, their inclusion produced no difference in the conclusions reached. These results are similar to the results obtained in the current analyses and thus inferences were unchanged.

It is unlikely that potential bias due to indexing procedures had operated differentially in the three countries. Hence, the role of epidemiology in influential papers dealing with clinical medicine, assessed for its involvement as a research method and/or as resource for scientific research, assumed importance to a variable degree in the three countries. This may reflect a wide array of inter-country differences in resources as well as in clinical, research, funding requirements, and publication practices affecting the literature output in the field of clinical medicine.

The criteria based on content and publication type are distinctive in that “content” identifies papers having some type of epidemiological involvement irrespective of study design including both observational and experimental types of epidemiological studies, whereas “publication type” identifies only the experimental type of epidemiological studies. Proportional differences between papers in the US and the UK were detectable (marginally significant) by the “content” criterion but almost negligible (non-significant) by the “publication type” criterion. This may indicate a higher level of utilization of epidemiology in clinical medicine in the US irrespective of study design, or conversely, a relatively high emphasis on the experimental type of epidemiological studies in the UK.

The authors’ affiliations in epidemiology or other closely related disciplines were significantly different among the three countries. This may indicate differences in the availability of human resources, collaboration practices, or both. The difference between the US and the UK was particularly interesting in view of the widely accepted notion that the two countries have heretofore taken leads in the historical development of epidemiology^4^^)^. In contrast, the Japanese medical community has a relatively shorter history of epidemiology and the importance of the latter in clinical medicine might not have been fully appreciated. Such a situation in Japan has recently been characterized as “a society where epidemiologists are ill respected^5^^)^.” However, the affiliation information may be biased, reflecting differences in the size of organizations and/or customs to express detailed departmental affiliation. If the differences are real, the finding suggests potential room for more collaboration with epidemiologists among clinical researchers in both the UK and Japan.

The increasingly wider gaps in recent years between the three countries in the proportion of the most frequently cited clinical papers with epidemiological involvement, and hence increasingly wider gaps in the emphasis of epidemiological involvement, is a conceivable characterization of the international literature in clinical medicine. Such a differential trend is unlikely to have been affected by changes in the practice of indexing articles. These are important findings, if it is accepted that epidemiology has “a key position in clinical evidence-based health sciences^6^^)^.”

Clinical research papers in the US had the highest proportion of epidemiological involvement and also the highest number of citations, whereas Japanese papers had the lowest proportion of epidemiological involvement and also the lowest number of citations. If the frequency of citation is indicative of the quality of the paper (i.e., objectivity, validity or credibility), then one interpretation is that the involvement of epidemiology enhances the quality of the paper. In the US, the involvement of epidemiology and/or biostatistics has become a standard requirement in many major proposals for funding in clinical research, although the extent of reference to epidemiology depends on the nature of the study, and varies from investigation to investigation. Based on the international comparison presented in the present paper, the requirement for epidemiologic considerations appears to have been rightfully justified.

In conclusion, the most frequently cited papers in clinical medicine from the US, the UK, and Japan were compared in terms of epidemiological involvement. When pre-determined criteria were applied based on the indexing of relevant MeSH keywords and publication type, in addition to the identification of relevant terms describing affiliation of authors, the involvement of epidemiology in clinical medicine was most apparent in the US, followed by the UK, but very weak in Japan. There was also an increasing trend in the proportion of clinical papers with epidemiological involvement in recent years in all three countries, but the trend was much stronger in the US than in either the UK or Japan.
